# Supplementary material for: Changes in Gut Microbiota after a Four-Week Intervention with Vegan vs. Meat-Rich Diets in Healthy Participants: A Randomized Controlled Trial
Source: Microorganisms. 2021 Mar 31;9(4):727. doi: 10.3390/microorganisms9040727 (PMC8066554; doi:10.3390/microorganisms9040727)
Supplement: Supplementary file 1 [file microorganisms-09-00727-s001.zip › Supplement_Figures.docx]

**Figure S2.** Heatmap of all ASVs with a significant interaction between diet and time, “_NA” describes unspecified species. Abundance values are log transformed and standardized. The upper block shows all ASVs that were depleted in VD and enriched in MD. The lower block shows all ASVs enriched in VD and depleted in MD (p_adj_ < 0.05). The green box emphasizes the remaining significant ASVs after filtering ASVs observed in at least 40% of samples.

**Figure S6.** PCoA with weighted UniFrac distances. Phylo1 and Phylo2 separate along Axis 1.
